# Effect of small interference RNA (siRNA) for *ADAMTS5 *on intervertebral disc degeneration in the rabbit anular needle-puncture model

**DOI:** 10.1186/ar2851

**Published:** 2009-11-04

**Authors:** Shoji Seki, Yumiko Asanuma-Abe, Koichi Masuda, Yoshiharu Kawaguchi, Kunihiro Asanuma, Carol Muehleman, Akiko Iwai, Tomoatsu Kimura

**Affiliations:** 1 Department of Orthopaedic Surgery, Faculty of Medicine, University of Toyama, 2630 Sugitani, Toyama, Toyama 930-0194, Japan; 2 Department of Orthopedic Surgery, Rush Medical College at Rush University Medical Center, 1653 W Congress Parkway, Chicago, IL 60612, USA; 3 Department of Orthopaedic Surgery, School of Medicine, University of California, San Diego, 9500 Gilman Drive, Mail Code 0863, La Jolla, CA 92093-0863, USA; 4 Department of Biochemistry, Rush Medical College at Rush University Medical Center, 1653 W Congress Parkway, Chicago, IL 60612, USA

## Abstract

**Introduction:**

The etiology of degenerative disc disease is unknown. Several investigators have reported the presence of proteolytic enzymes, such as the matrix metalloproteinase (MMP) and ADAMTS (a disintegrin and metalloprotease with thrombospondin-like repeats) families, in degenerated human discs. Glasson and colleagues recently reported that a significant reduction occurs in the severity of cartilage destruction in *ADAMTS5 *knockout mice compared with wild-type mice. The purpose of this study was to evaluate the suppressive effects of injections of *ADAMTS5 *small interference RNA (siRNA) oligonucleotide on intervertebral disc degeneration in the rabbit anular needle-puncture model.

**Methods:**

Rabbit nucleus pulposus (NP) cells were transfected with siRNA oligonucleotides specific for *ADAMTS5 *or the control. The suppression of the *ADAMTS5 *gene by siRNA transfection was assessed by using real-time polymerase chain reaction (PCR), both in monolayer and alginate bead cultures with or without interleukin-1β (IL-1β) stimulation. The effect of siRNA was determined *in vivo *by using the rabbit anular needle-puncture model (control group: n = 8; ADAMTS5 group: n = 8). One week after the initial anular puncture, the animals received an injection of the control or anti-*ADAMTS5 *oligonucleotide (100 μg each at the L2/3 and L4/5 level; 16 discs/group). Disc height, magnetic resonance imaging (MRI) (Thompson classification and signal intensity), and safranin-O staining (histologic grade) were assessed.

**Results:**

IL-1β treatment significantly increased the *ADAMTS5 *mRNA level in NP cells (*P *< 0.01). *ADAMTS5 *gene suppression was 70% compared with the control oligonucleotide in both monolayer and alginate bead culture with or without stimulation with IL-1β. The injection of anti-*ADAMTS5 *oligonucleotide *in vivo *resulted in improved MRI scores with increased signal intensity and improved histologic grade scores with statistical significance (*P *< 0.05). No significant change in disc height was observed.

**Conclusions:**

A single injection of *ADAMTS5 *siRNA induced the suppression of degradation in NP tissues, as shown by significantly improved MRI and histologic grades. The mechanism of response to siRNA may be worthy of exploration for possible therapeutic purposes.

## Introduction

The intervertebral disc (IVD) encompasses two structures, the anulus fibrosus (AF) and the nucleus pulposus (NP). The predominant matrix component of the AF is collagen type I, whereas the NP contains randomly organized collagen fibers (mainly type II) and highly hydrated proteoglycans, primarily aggrecan, which impart compressive resistance to the tissue. Aggrecan is cleaved at a specific "aggrecanase" site [1,2]; this cleavage results from the action of several members of the ADAMTS (adisintegrin and metalloprotease with thrombospondin-like repeats) family [3-6]. In a murine model of osteoarthritis, *ADAMTS5*-knockout mice have a significantly reduced level of cartilage destruction, compared with wild-type mice [7]. Glasson and colleagues [8] also reported that no effect in *ADAMTS4 *knockout mice was noted on the progression or severity of osteoarthritis after surgical induction of joint instability. However, the dual deletion of *ADAMTS4 *and *ADAMTS5 *provided significant protection against proteoglycan degradation *ex vivo *and decreased the severity of murine osteoarthritis *in vivo *[9].

Compared with cartilage, the NP has a higher content of aggrecan that is more degraded and a higher proportion of molecules not bound to hyaluronan [10]. A recent human cadaveric study revealed the presence of aggrecanase-generated aggrecan fragments and abundant levels of ADAMTS5 in human IVDs, regardless of the level of disc degeneration, based on magnetic resonance imaging (MRI) grade classification [11]. This study also showed that larger quantities of ADAMTS4 are present in human NP and AF tissues derived from discs with a greater level of disc degeneration (grade 4) compared with those from discs with lower level of disc degeneration (grade 2). Thus, it remains unclear whether *ADAMTS4 *or *ADAMTS5 *is the major aggrecanase responsible for degradation of aggrecan in the human IVD. Modulating the enzymatic activity or gene expression of the responsible enzymes might be a valid approach for protecting human IVD tissues from degradation.

IVDs of patients with lumbar disc herniation have been shown to express proinflammatory cytokines, such as interleukin-1β (IL-1β) and tumor necrosis factor-α (TNF-α) [12,13], which are known to stimulate the expression of ADAMTS in bovine cartilage [14,15]. The regulation of ADAMTS4 and ADAMTS5 has been reported to differ slightly. For example, although a highly selective inhibitor of IκB kinase did not inhibit the secretion of ADAMTS4, it blocked ADAMTS5 secretion in the same concentration range that inhibited aggrecan degradation in bovine cartilage [15]. Furthermore, whereas *ADAMTS5 *mRNA was expressed in human normal and OA cartilage [16], *ADAMTS4 *mRNA was very low *in vivo *and was induced *in vitro *only after stimulation with IL-1β. These results suggest that investigating both *ADAMTS4 *and *ADAMTS5 *may shed light on the mechanism of IVD degeneration.

Biologic treatment strategies for human IVD degeneration include increasing the levels of anabolic growth factors or blocking the catabolic cascade or both. On the anabolic side, an *in vivo *rabbit anular puncture model of disc degeneration showed the anabolic effects of bone morphogenetic protein-7 [17] and growth and differentiation factor-5 [18]. The anticatabolic effects of factors, such as caspase inhibitor [19], tissue inhibitor of metalloproteinase-1 [20], IL-1 receptor, anti-TNF-α antagonists [12,21,22], and others have been shown on the extracellular matrix metabolism of IVD cells *in vitro*.

The anticatabolic role of the specific inhibition of *ADAMTS4 *and *ADAMTS5 *in human cartilage was recently found by using a small interfering RNA (siRNA) approach in normal and osteoarthritic explants [23]. To date, no report has been made that an anticatabolic factor suppresses IVD degeneration *in vivo*. The purpose of this study was to prove our hypothesis that a single injection of *ADAMTS5 *siRNA inhibits the production of ADAMTS5 and suppressed IVD degeneration in the rabbit anular needle-puncture model.

## Materials and methods

### *In vitro *study

#### Cell preparation and alginate bead culture

Lumbar IVDs from four consecutive levels (L2/3, L3/4, L4/5, and L5/S1) were dissected from the spines of adolescent Japanese white rabbits (1 to 1.5 kg, 6 to 9 weeks old) after killing by injection of an excess amount of sodium pentobarbital (Dainippon Pharmaceutical, Osaka, Japan). Tissues were separately harvested from the NP and the outer layer of the AF, and the cells were isolated by using a sequential proteinase and collagenase digestion, as previously described [24]. Primary cells were expanded in monolayer culture in complete media (Dulbecco's Modified Eagles Medium (DMEM) supplemented with 10% FBS, 100 U/ml penicillin, 100 μg/ml streptomycin, and 0.5 μg/ml amphotericin B (Fungizone)). After one passage, cells were suspended in sodium alginate (1.2% solution in 155 m*M* NaCl; Cambrex CC-3234, Charles, Iowa, USA) at a density of 5 × 10^5 ^cells/ml. The beads were maintained for up to 14 days with Chondrocyte Differentiation Media (Cambrex CC-3225) and seeded in a 12-well plate at a density of 1 × 10^5 ^cells/well.

### Establishment of siRNA for *ADAMTS5 *oligonucleotide and transient transfection

The siRNA oligonucleotide for the rabbit *ADAMTS5 *gene was constructed from a completely homologous region of sequences in the *ADAMTS5 *gene of the human, rat, and mouse from the NCBI website [25]. The reverse transcriptase-polymerase chain reaction (RT-PCR) primers were constructed from this homologous region, and RT-PCR was completed. PCR products were collected, and the rabbit *ADAMTS5 *gene was cloned by using the TA Cloning Kit (Invitrogen, Carlsbad, CA, USA) and confirmed by sequencing by using the ABI PRISM 310. Sequences for primers used in these analyses were as follows: 5'-CTCCCAGGACAAACCTACGA-3' and 5'-CCTCTTCCCTGTG CAGTAGC-3' for *ADAMTS5 *cDNA amplification. SiRNA for the *ADAMTS5 *oligonucleotide was constructed by using the Takara Website [26]. Sequences for the control and *ADAMTS5 *oligonucleotides of the siRNA used in these analyses were as follows: sense oligonucleotide 5'-CGAUCCUCAAAGCACUACUTT-3', anti-sense oligonucleotide 5'-AGUA GUGCUUUGAGGAUCGTT-3' for the control, sense oligonucleotide 5'-CCACCAUCACG GAAUUCCUTT-3', and anti-sense oligonucleotide 5'-AGGAAUUCCGUGAUGGUGGTT-3' for *ADAMTS5*. Sense and antisense oligonucleotide siRNAs were separately dimerized for the control and *ADAMTS5*.

### Confirmation of the knockdown rate of the *ADAMTS5 *GENE with or without IL-1β stimulation in monolayer culture

A standard for the rabbit *ADAMTS5 *gene was constructed from the sequence originally analyzed. For transient transfection, NP cells were seeded in a 12-well plate at a density of 1 × 10^5 ^cells/well and cultured in complete media.

#### Effect on constitutive expression

After 48 hours, the NP cells were transiently transfected with the anti-*ADAMTS5 *oligonucleotide or control oligonucleotide, added directly to media without transfection reagents. After 48 hours' incubation, the cells were harvested, and the mRNA level of *ADAMTS5 *was assessed.

#### Effect on IL-1β-stimulated expression

After 48-hour preculture, cells were cultured with or without IL-1β (10 ng/ml) (Roche, Mannheim, Germany) in serum-free DMEM. After 24 hours of IL-1 treatment, anti-*ADAMTS5 *oligonucleotide or control oligonucleotide was added to the culture for transfection. After 24 hours' incubation with siRNA, NP cells were collected and subjected to mRNA analysis.

### Confirmation of the knockdown rate of the *ADAMTS5 *gene by "siSTABLE" siRNA in alginate bead culture

Freshly prepared stable anti-*ADAMTS5 *oligonucleotide (Dharmacon siSTABLE, Thermo Scientific, Lafayette, CA, USA) was prepared for *in vivo *experiments, but first tested in an *in vitro *alginate culture system. NP cells were cultured in alginate beads, as described earlier. After 14 days, NP cells in alginate beads were transfected with anti-*ADAMTS5 *oligonucleotide or control oligonucleotide (Dharmacon) without transfection reagents. Results are reported normalized to *GAPDH*.

### RNA isolation and real-time PCR

Total RNA was extracted from transfected cells by using Isogen (Nippongene, Tokyo, Japan) and purified with the SV Total RNA Isolation System (Promega, Madison, WI, USA).

Random-primed cDNAs were synthesized by using Multiscribe reverse transcriptase (PE Applied Biosystems, Foster, CA, USA). Quantitative real-time PCR was carried out by using a PRISM 7700 sequence detector with the QuantiTect SYBR Green PCR kit (Qiagen, Valencia, CA, USA) according to the manufacturer's instructions. The relative expression of *ADAMTS5 *was calculated by using the comparative threshold (C_T_) method, as previously described [27]. Results are reported normalized to the housekeeping gene glyceraldehyde 3-phosphate dehydrogenase (*GAPDH*).

### *In vivo *study

#### Establishment of a degenerative IVD by using the rabbit anular needle-puncture model and injection of the anti-*ADAMTS5 *oligonucleotide

An anular puncture model was established by using an 18-gauge needle at a defined depth of puncture (5 mm), as previously reported [28]. New Zealand white rabbits (n = 12), weighing approximately 3.5 to 4.0 kg (5 months old), were used in this study with the approval of the Institutional Animal Care and Use Committee (06-067). Under general anesthesia, lumbar IVDs were exposed, and the initial puncture with an 18-gauge needle was performed on two noncontiguous discs (L2/3 and L4/5), with the disc (L3/4) between the punctured discs left intact as a control [17]. One week after the initial puncture, the discs were exposed again from the contralateral side, and either control siRNA or *ADAMTS5 *siRNA oligonucleotide (Dharmacon, was injected into the center of the NP by using a 26-gauge needle (10 μg in 10 μl phosphate-buffered saline (PBS) per disc). The timing of injection was earlier than that used in other studies [17,18] to reveal the effect of an injection of *ADAMTS5 *siRNA during the acute phase of disc degeneration. Nine weeks after the initial anular puncture (8 weeks after the injection), all rabbits were killed.

#### Radiographic analysis of disc height

Radiographs were taken at time 0 and at weeks 1, 2, 3, 5, 7, and 9 after the puncture. Extreme care was taken to maintain a consistent level of anesthesia during radiography of each animal at each time point to obtain a similar degree of muscle relaxation, which may affect the disc height. Therefore, the preoperative radiograph was always used as a baseline measurement. Radiographs were digitally scanned and digitally stored by using an image-capture software program.

#### Image analysis

All radiographic images were independently analyzed by using a custom program for MATLAB software (Natick, MA, USA) by an orthopedic researcher who was blinded to the treatment groups. Data are reported as the IVD height expressed as the disc-height index (DHI) (DHI = intervertebral disc height/adjacent vertebral body height) [17]. Changes in the disc-height index of injected discs were expressed as percentage DHI (%DHI) and normalized to the measured preoperative intervertebral disc height (%DHI = (Postoperative DHI/Preoperative DHI) × 100) [17]. To avoid the influence of anesthesia, the %DHI at the experimental level was further normalized to %DHI at the nonpunctured level (normalized %DHI = (Punctured %DHI/Nonpunctured %DHI) × 100).

#### MRI assessment

MRI examinations were performed on all rabbits in the study by using a 0.3-T imager (Airis II, version 4.0 A; Hitachi Medical System America, Inc., Twinsburg, Ohio, USA) with a quadrature extremity coil receiver. After killing, the spinal columns with surrounding soft tissue were isolated and subjected to MRI analysis [17]. An observer, blinded to the study groups, used a modified Thompson classification based on changes in the degree and area of signal intensity from grade 1 to 4 (1 = normal, 2 = minimal decrease in signal intensity but obvious narrowing of high-signal area, 3 = moderate decrease in signal intensity, and 4 = severe decrease in signal intensity) to evaluate the MRIs. The intraobserver and interobserver reliability correlation coefficients of MRI grading based on two evaluations were excellent (K = 0.98, 0.90, respectively), as determined by the Cohen kappa correlation coefficient [28].

#### Histologic evaluations

After MRI assessment, the experimental IVDs were excised from the vertebral body-disc-vertebral body unit, and each IVD was fixed in 10% formalin, decalcified, embedded in paraffin, sectioned, and assessed with conventional histology and immunostaining. Midsagittal sections (5 μm) of each IVD were stained either with hematoxylin and eosin or with safranin-O. An observer, blinded to the experiment, analyzed the histologic sections and graded them by using our recently established protocol [28].

### Statistics

The %DHI was statistically analyzed by using a two-way repeated analysis of variance and Fisher protected least significant difference as a *post hoc *test (factors; time and treatment group). Other statistical analyses were assessed with the Mann-Whitney *U *test. *In vitro *experiments were performed 3 times.

## Results

### *In vitro *study

Rabbit NP cells were chosen because their response to IL-1β was more consistent than that of AF cells (as determined from previous experiments) and because the injection site in the NP has a higher concentration of proteoglycans. The effectiveness of administration of *ADAMTS5 *siRNA might be influenced by the rich positively charged matrix in NP tissues.

#### Effect of siRNA on rabbit *ADAMTS5 *gene expression in rabbit NP cells cultured in monolayer with or without stimulation with IL-1B

**Figure 1:**
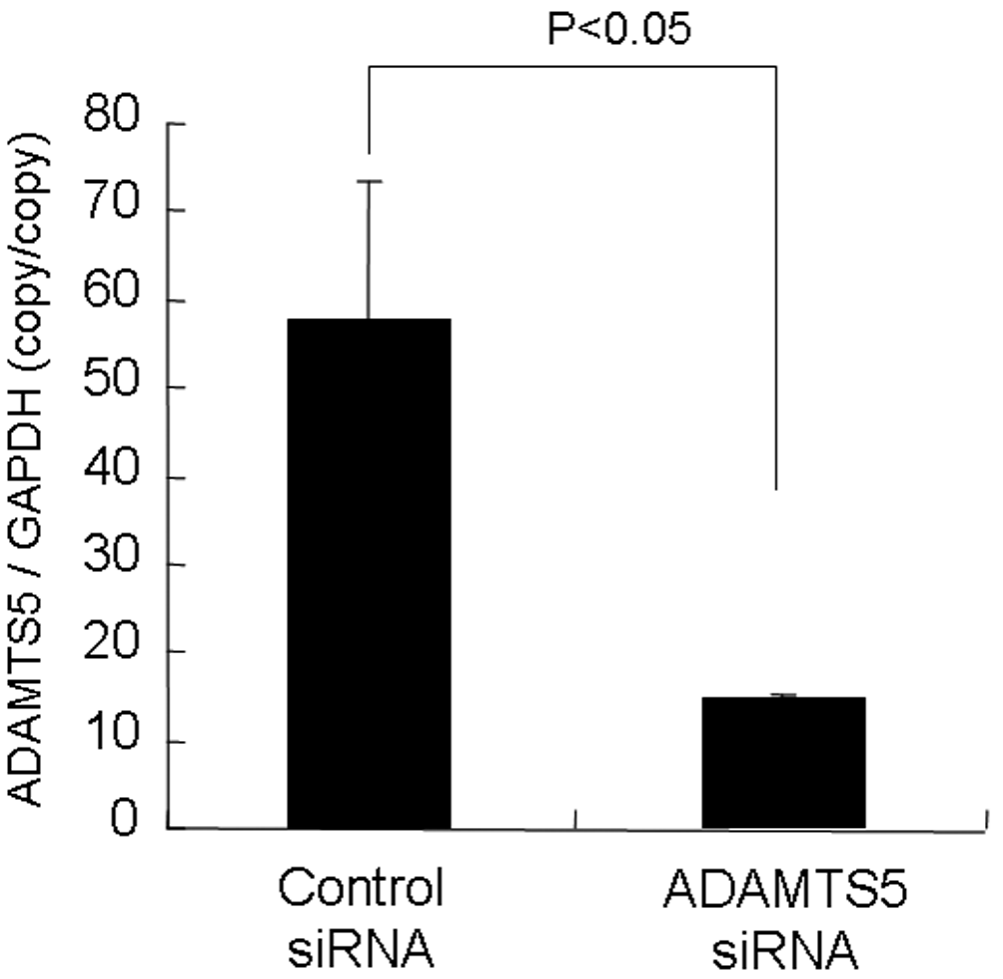
Establishment of small interfering RNA (siRNA) oligonucleotide for *ADAMTS5 *in rabbit nucleus pulposus (NP) cells. After the 48-hour preculture period, rabbit NP cells were transfected with the siRNA oligonucleotide specific for either the control or *ADAMTS5*. At 48 hours after transfection in NP cells, the *ADAMTS5 *siRNA-transfected cells showed approximately a 75% knock-down of *ADAMTS5 *mRNA compared with the control siRNA. The results are reported normalized to glyceraldehyde 3-phosphate dehydrogenase (*GAPDH*).

**Figure 2:**
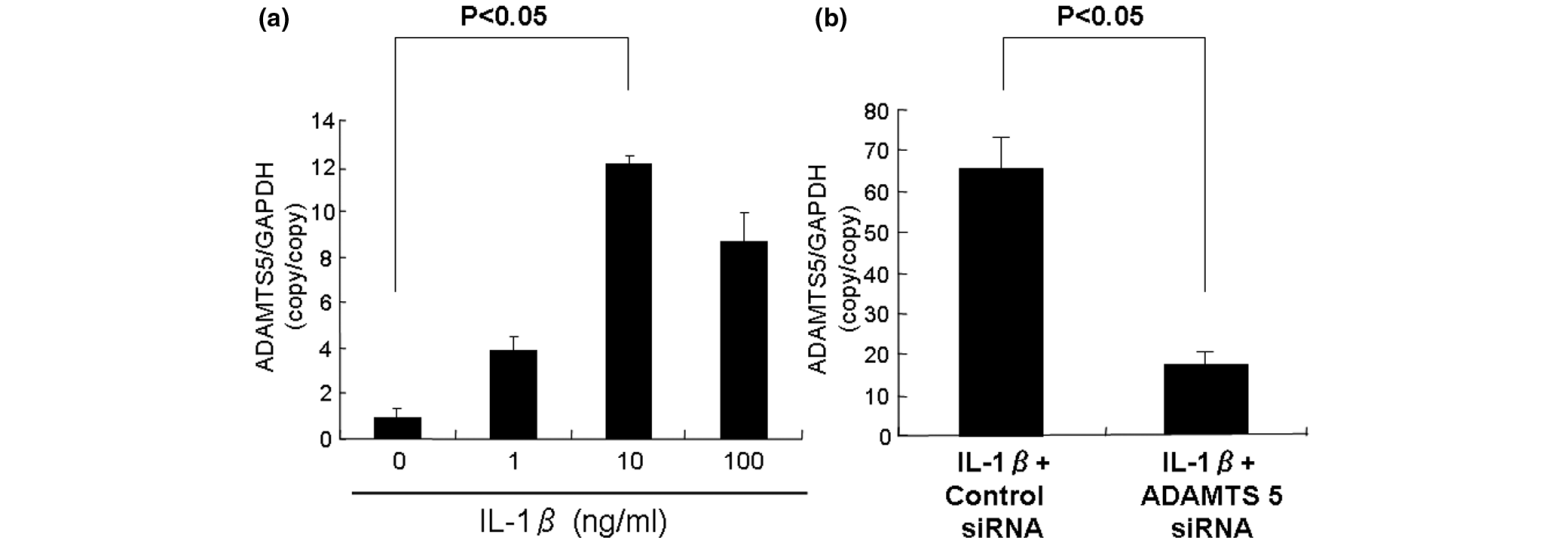
Effect of interleukin-1β (IL-1β) stimulation on *ADAMTS5 *mRNA expression in rabbit nucleus pulposus (NP) cells. After real-time polymerase chain reaction (PCR), the *ADAMTS5 *mRNA expression level after IL-1β stimulation (24 hours) in rabbit NP cells is shown **(a)**. IL-1β at 10 ng/ml induced the highest level of increased expression of mRNA for *ADAMTS5 *(about 12-fold); that concentration was chosen for subsequent studies. **(b) **NP cells seeded in a 12-well plate at a density of 1 × 10^5 ^cells/well. After the 48-hour preculture period, cells were cultured in serum-free media in the presence of IL-1β (10 ng/ml) for 24 hours. After the 24-hour treatment with IL-1β, NP cells were transiently transfected with the anti-*ADAMTS5 *oligonucleotide or control oligonucleotide by adding oligonucleotide directly to the culture media. Twenty-four hours later, NP cells were collected, and the expression of *ADAMTS5 *was analyzed with real-time PCR. *ADAMTS5 *mRNA expression was knocked down by about 70% in rabbit NP cells that were transfected with *ADAMTS5 *siRNA and stimulated with IL-1β (10 ng/ml). The results are reported normalized to *GAPDH*.

We confirmed that the siRNA oligonucleotide we constructed knocked down the *ADAMTS5 *gene in rabbit NP cells. At 48 hours after transfection, the NP cells that received the *ADAMTS5 *siRNA oligonucleotide showed approximately a 75% knockdown of constitutive expression of *ADAMTS5 *mRNA (Figure 1). This suppression was observed in all three experiments. Real-time PCR revealed that IL-1β treatment for 24 hours increased the abundance of mRNA for *ADAMTS5 *(about 12-fold) in a dose-dependent manner in rabbit NP cells (Figure 2a). Based on these results, a concentration of IL-1β of 10 ng/ml was chosen for further studies. Subsequently, after IL-1β treatment at 10 ng/ml for 24 hours, NP cells were transfected with *ADAMTS5 *and control siRNA. At 24 hours after the transfection, the abundance of *ADAMTS5 *mRNA was knocked down by 70% compared with the control group in rabbit NP cells (Figure 2b). This effect persisted for 2 weeks (data not shown).

#### Effect of adamts5 oligonucleotide on rabbit NP cells cultured in alginate beads

**Figure 3:**
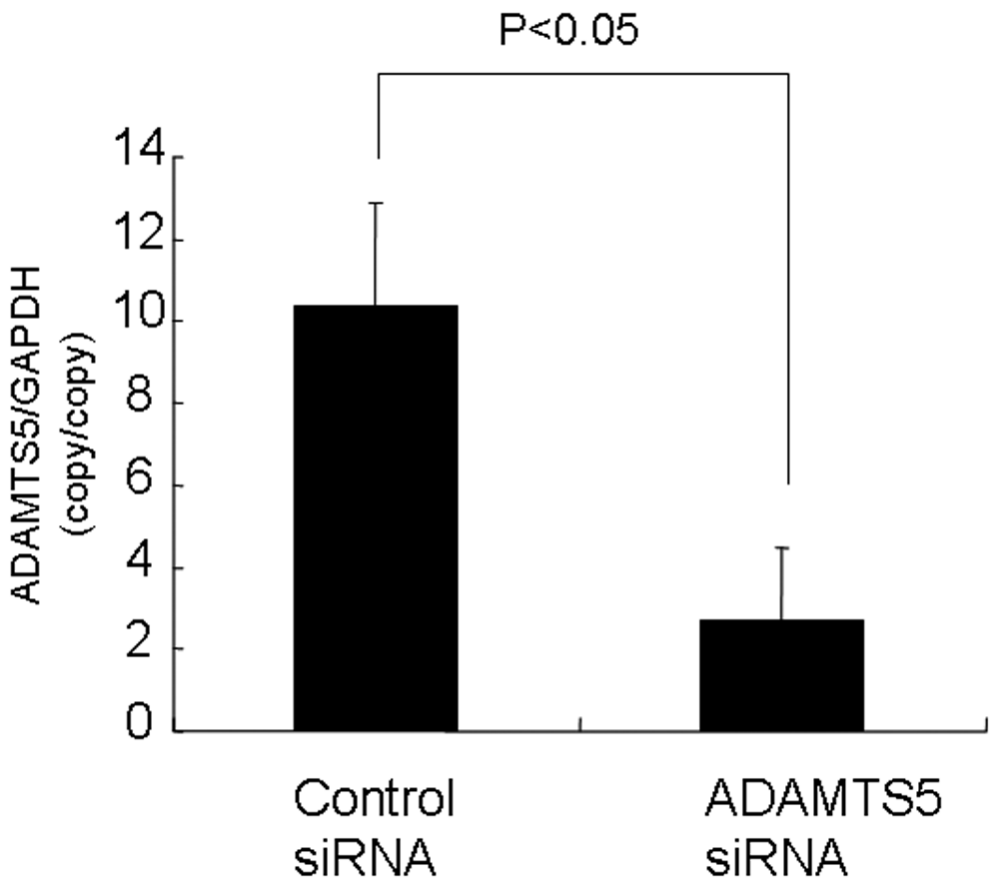
Effect of *ADAMTS5 *oligonucleotide on rabbit nucleus pulposus (NP) cells in alginate bead culture. NP cells were suspended in sodium alginate at a density of 5 × 10^5 ^cells/ml and maintained for up to 14 days. After 14 days, the NP cells in alginate beads were transfected with anti-*ADAMTS5 *oligonucleotide or control oligonucleotide without using gene-delivery reagents. In NP cells in alginate bead culture, the expression of the *ADAMTS5 *gene was approximately 70% of that seen with the control oligonucleotide. The results are reported normalized to *GAPDH*.

It is possible that the injected siRNA cannot penetrate the matrix of the target tissue and induce metabolic changes. To confirm our hypothesis that *ADAMTS5 *siRNA has an effect in a three-dimensional environment, the efficacy of *ADAMTS5 *was tested by using the alginate bead-culture system. We confirmed that the *ADAMTS5 *gene was significantly knocked down by using *ADAMTS5 *siRNA with no transfection reagent. The knockdown rate of the *ADAMTS5 *siRNA cells was 70% (Figure 3); this effect also persisted for about 2 weeks (data not shown).

### *In vivo *Study

#### Radiographic assessment

**Figure 4:**
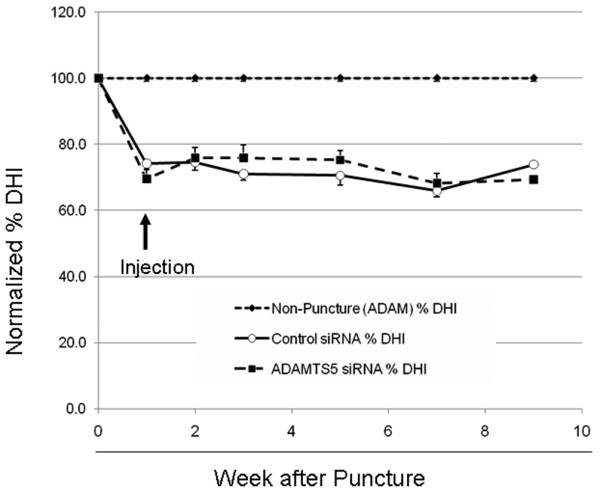
Radiographic assessment in the rabbit anular puncture model of disc degeneration. An anular puncture model was established in 5-month-old New Zealand white rabbits. Under general anesthesia, lumbar intervertebral discs were exposed, and the initial puncture with an 18-gauge needle at a defined depth of puncture (5 mm) was performed on two noncontiguous discs (L2/3 and L4/5), with the disc (L3/4) between the punctured discs left intact as a control. One week after the initial puncture, either control small interfering RNA (siRNA) or *ADAMTS5 *siRNA oligonucleotide (10 μg in 10-μl phosphate-buffered saline (PBS) per disc) was injected into the center of the nucleus pulposus by using a 26-gauge needle. Nine weeks after the initial anular puncture (8 weeks after the injection), all rabbits were killed. Radiographs were taken at time 0 and at weeks 1, 2, 3, 5, 7, and 9 after the puncture to quantity changes in the disc-height index (DHI). The %DHI was calculated as [%DHI = (Postoperative DHI/Preoperative DHI) × 100]. At 8 weeks after the *ADAMTS5 *siRNA injection, no difference was found in the mean %DHI of the *ADAMTS5 *siRNA-injected punctured discs compared with the punctured discs that received the control siRNA injection (*P *> 0.05, repeated ANOVA).

Radiographic assessments were performed to confirm that the rabbits received identical punctures with an 18-gauge needle at the correct levels. The needle puncture was found to have reduced the %DHI at 1 week (70% DHI; *P *> 0.06, Figure 4), with no significant difference in %DHI between the two groups. At that time, the control oligonucleotide or anti-*ADAMTS5 *oligonucleotide (100 μg in 10 μl of PBS) was administered into the NP of the rabbit IVDs. After the siRNA injections, no significant differences were found in the %DHI between the control siRNA and the *ADAMTS5 *siRNA groups during the observation period (Figure 4).

#### MRI analysis

**Figure 5:**
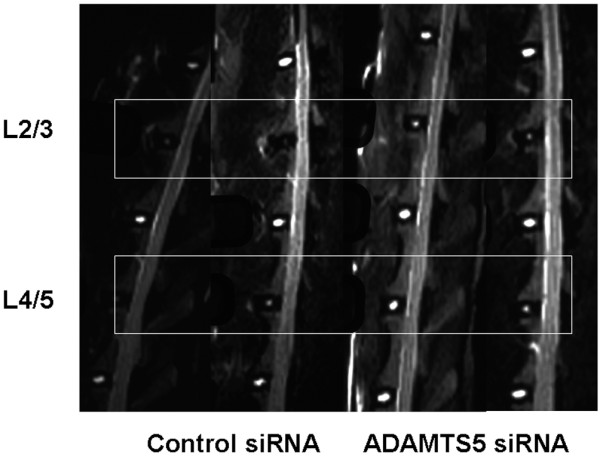
Magnetic resonance imaging (MRI) findings after small interfering RNA (siRNA) oligonucleotide injection in the rabbit anular puncture model of disc degeneration. MRI examinations were performed on all spinal columns isolated from the rabbits *ex vivo *at death 8 weeks after the siRNA oligonucleotide injection. In these representative MRIs, the T_2 _signal intensity in the nucleus pulposus of the *ADAMTS5 *siRNA-injected discs was stronger than that in the control siRNA-injected discs.

**Figure 6:**
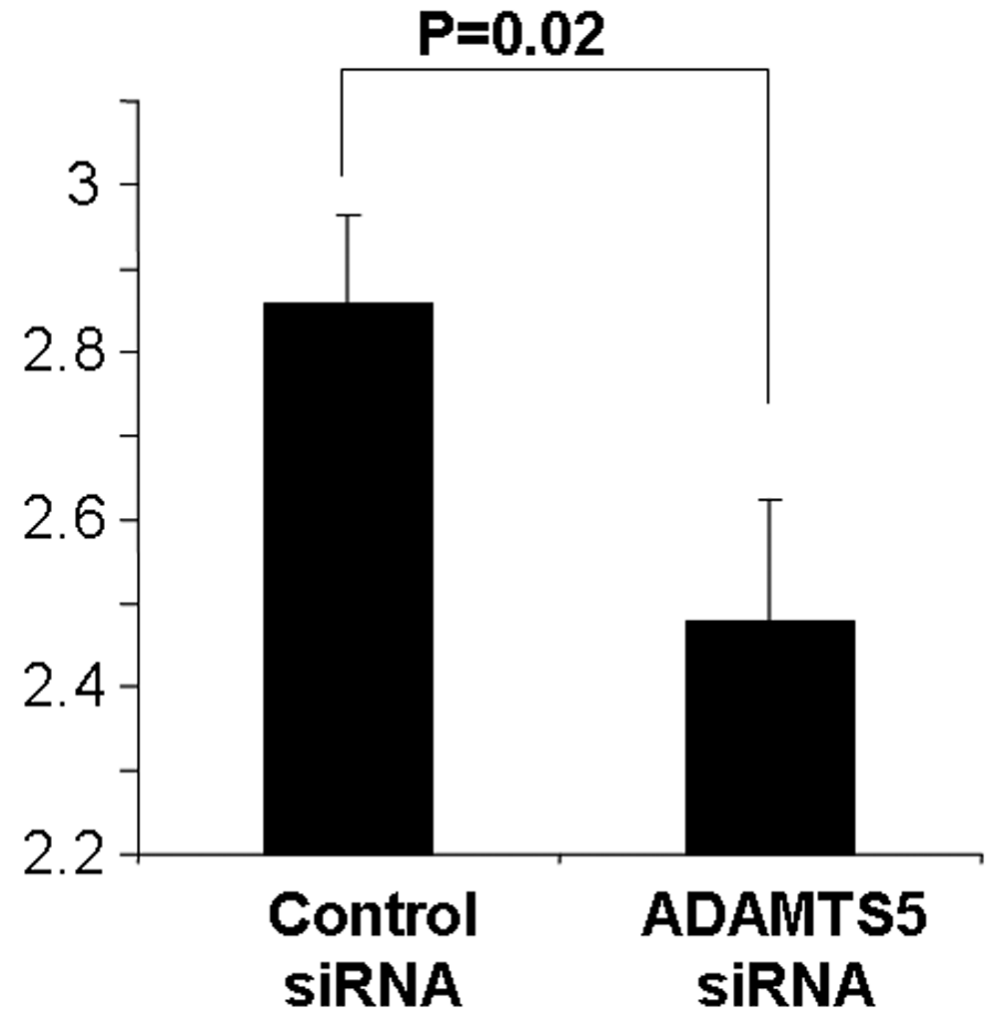
Magnetic resonance imaging (MRI) assessment 8 weeks after small interfering RNA (siRNA) injection in the rabbit anular puncture model of disc degeneration. An observer blinded to the study assessed the MRIs by using the modified Thompson scale, based on changes in the degree and area of signal intensity from grades 1 to 4. After assessment of the MRI grades, a significantly lower (better) MRI grade in the *ADAMTS5 *siRNA-injected discs was observed compared with the control siRNA-injected discs (*P *= 0.02, Mann-Whitney test).

MRI analysis was performed at 8 weeks after the siRNA injections when the animals were killed. The MRI of the NP in the *ADAMTS5 *siRNA group showed a stronger T_2 _signal intensity than that found in the Control group (Figure 5). When disc degeneration was assessed by using the Thompson MRI grading score, the grading score was significantly lower (better) in the *ADAMTS5 *siRNA group than in the control siRNA group (*P *= 0.02, Mann-Whitney *U *test) (Figure 6).

#### Histologic evaluation

**Figure 7:**
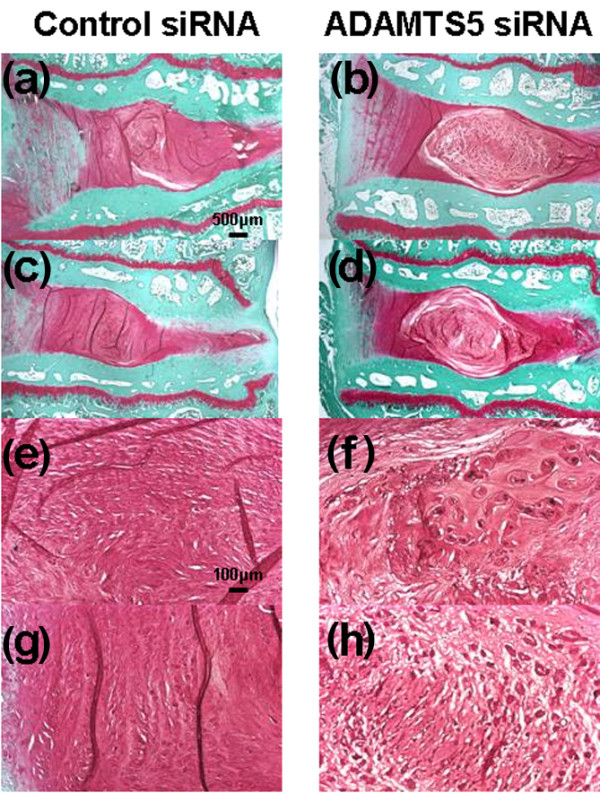
Safranin-O-stained sections reflecting typical histologic changes after injection of control small interfering RNA (siRNA) or *ADAMTS5 *siRNA in the rabbit anular puncture model of disc degeneration. Eight weeks after the control or *ADAMTS5 *siRNA injections, the control siRNA group displayed a complete loss of nucleus pulposus (NP) tissues, which had been replaced by a fibrocartilaginous tissue **(a, c)**. The severely degenerated discs that had received the control siRNA showed a loss of proteoglycans and the collapsed, wavy fibrocartilage lamellae typical of the anulus fibrosus (AF), with associated fibrochondrocytes **(e, g)**. In the *ADAMTS5 *siRNA-injected discs, safranin-O staining demonstrated the maintenance of intervertebral disc structure with a lightly stained matrix and large cells **(b, d)**; the NP was rounded and bloated looking, and consisted of numerous large, vacuolated cells and smaller chondrocyte-like cells **(f, h)**. A clear demarcation was seen between the NP and inner anulus in the *ADAMTS5 *siRNA-injected discs. (Magnification ×20 (a-d), ×100 (e-h)). The level in a, b, e, and f is L2/3, and in c, d, g, and h is L4/5.

**Figure 8:**
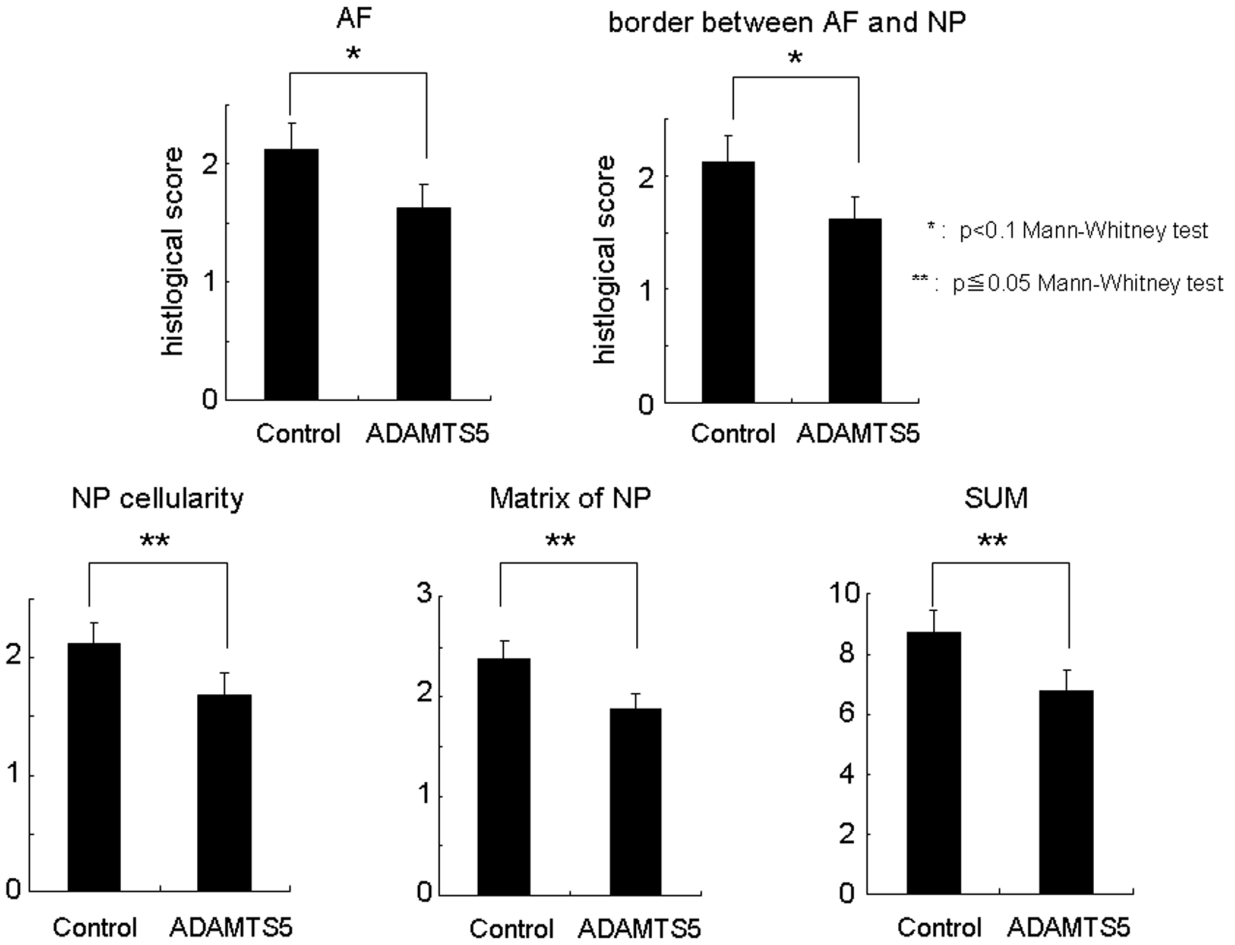
Histologic assessment after *ADAMTS5 *small interfering RNA (siRNA) or control siRNA injection in the rabbit anular puncture model of disc degeneration. In *ADAMTS5 *siRNA-injected discs, the anulus fibrosus (AF) and the border between the AF and nucleus pulposus (NP) showed a tendency to have a lower (better) histologic score than the control siRNA-injected discs (Mann-Whitney test; *P *< 0.1). In the NP, the cellularity, matrix, and total grading score (SUM) were significantly better in the *ADAMTS5 *siRNA-injected discs than in the control siRNA-injected discs (Mann-Whitney test; *P *< 0.05).

The injection of *ADAMTS5 *siRNA significantly affected the histochemical changes found with IVD degeneration. Eight weeks after the control or *ADAMTS5 *siRNA injections, the control siRNA group displayed a complete loss of NP tissues, which had been replaced by a fibrocartilaginous tissue (Figure 7a and 7c). The severely degenerated discs that had received the control siRNA showed a loss of proteoglycans and the collapsed, wavy fibrocartilage lamellae typical of the AF with associated fibrochondrocytes (Figure 7e and 7g). In the discs that received the *ADAMTS5 *siRNA, safranin-O staining demonstrated the maintenance of IVD structure with a lightly stained matrix and large cells (Figure 7b and 7d); the NP was rounded and well filled with numerous large, vacuolated cells and smaller chondrocyte-like cells typical of the normal IVD (Figure 7f and 7h). The histologic grading scores demonstrate that the cellularity and matrix of the NP in the *ADAMTS5 *siRNA-treated discs were significantly lower (better) than those in the control siRNA group (Figure 8). The total grading score (SUM) in the *ADAMTS5 *siRNA-treated discs was also significantly lower than those in the control siRNA group (Mann-Whitney; *P *< 0.05) (Figure 8). The scores for the AF and the border between the AF and NP in the *ADAMTS5 *siRNA-treated discs showed a trend to be lower than those in the control discs (Mann-Whitney; AF; *P *= 0.06; border between the AF and NP; *P *= 0.08).

## Discussion

With the rabbit anular puncture model, this study explored the efficacy of a direct injection of *ADAMTS5 *siRNA into the NP on the delay or attenuation of disc degeneration. Our results demonstrate that the designed *ADAMTS5 *siRNA was (a) active *in vitro *and (b) effective in suppressing the degeneration of the NP tissue in the *in vivo *rabbit model. However, the injection of *ADAMTS5 *siRNA did not induce the anticipated recovery of disc height.

We successfully designed and constructed an siRNA oligonucleotide with biologic activity for the rabbit *ADAMTS5 *gene. *ADAMTS5 *siRNA-transfected rabbit NP cells showed approximately a 75% knockdown of *ADAMTS5 *mRNA compared with the control siRNA. Although we demonstrated that IL-1β treatment significantly increased the *ADAMTS5 *mRNA level in NP cells, the suppression of the expression of the *ADAMTS5 *gene by *ADAMTS5 *siRNA was 70% compared with the control oligonucleotide in both monolayer and alginate bead culture under stimulation with IL-1β.

The intradiscal injection of *ADAMTS5 *siRNA during the acute phase of disc degeneration after anular puncture in the rabbit delayed the progression of disc degeneration, as assessed by MRI scores, signal intensity of NP on MRI, and histologic scores. MRI findings of a high T_2 _signal intensity in the NP indicate that the NP in the punctured discs treated with ADAMTS5 siRNA was hydrated. Therefore, ADAMTS5, which cleaves the core protein of aggrecan, may significantly contribute to the loss of water content of the NP after anular puncture. However, the reason that the disc height loss was not reversed by the injection of *ADAMTS *siRNA remains to be determined. One possible explanation is that the treatment with siRNA for *ADAMTS5 *is an anticatabolic one, not anabolic. In addition, a possibility remains that the injected siRNA was retained in the NP area, where the siRNA was injected, by some mechanism, or mainly internalized by NP cells and did not distribute to the AF area. In a previous study using the rabbit anular puncture model, the injection of osteogenic protein-1 into the NP induced an increased proteoglycan content of both the AF and the NP and the recovery of disc-height loss by 6 weeks [17]. One could speculate that the maintenance of disc height is determined by the structural integrity of the anulus, which could not be fully assessed through MRI and histology in a quantitative fashion. Furthermore, we did not test different doses of *ADAMTS5 *siRNA in the anular puncture model, nor did we assess the half-life of injected siRNA. The limited effects of *ADAMTS5 *siRNA may point to a complex involvement of multiple enzymes in disc degeneration. Nevertheless, the strong suppression of the *ADAMTS5 *gene by siRNA *in vitro *and *in vivo*, especially in NP tissues, indicates that ADAMTS5 might play an important role in IVD degeneration.

Histologic findings from safranin-O staining were supportive of the maintenance of NP tissues in the *ADAMTS5 *siRNA-treated discs, as observed with MRI. The improving histologic scores associated with the NP might indicate that ADAMTS5 is more involved in matrix degeneration of the NP than that in the AF. In addition, the direct injection of siRNA into the NP may induce a localized improvement. It is worth noting that the histologic scores for the AF and the border between the AF and NP in the *ADAMTS5 *siRNA-treated discs showed a trend to be lower (improved) when compared with those for the control discs. These findings might indicate that the inhibition of degeneration or improved reparative activity of the NP may have contributed to the improved histologic grading for the AF and the border between the AF and NP.

The treatment of human disc cells with IL-1 induced an imbalance between catabolic and anabolic events, responses that represent the changes seen during disc degeneration [12,13,29]. After treatment with IL-1, the aggrecanases (*ADAMTS4, 5*), matrix metalloproteinase-3 (*MMP-3*), and *MMP-13*, gene expression was increased in cells derived from the human NP cells [13]. Séguin and colleagues [30] reported that induction of *ADAMTS4 *and *-5 *mRNA occurred downstream of NF-κβ activation in NP cells. These results, and the recent reports on the contribution of IL-1 in disc degeneration [31], may indicate that as disc degeneration progresses, more ADAMTS5 is expressed in the IVD, with a high association with an increased amount of IL-1.

In summary, we have shown evidence that the suppression of *ADAMTS5 *in turn suppressed IVD degeneration; this suggests the possible contribution of ADAMTS5 to disc degeneration, especially in the NP of the rabbit anular puncture model of disc degeneration.

## Conclusions

A single injection of *ADAMTS5 *siRNA suppressed disc degeneration in the NP, as shown by the significantly improved MRI and histologic grades. The results may suggest that ADAMTS5 contributes to the degeneration of NP tissues in the rabbit anular puncture disc-degeneration model. The mechanism for the differences in response to siRNA in disc height and MRI findings may be worthy of exploration.

## Abbreviations

ADAMTS: a disintegrin and metalloprotease with thrombospondin-like repeats; AF: anulus fibrosus; C_T_: comparative threshold; DHI: disc height index; GAPDH: glyceraldehyde 3-phosphate dehydrogenase; IL-1β: interleukin-1β; IVD: intervertebral disc; MMP: matrix metalloproteinase; MRI: magnetic resonance imaging; NP: nucleus pulposus; PBS: phosphate-buffered saline; PCR: polymerase chain reaction; RT-PCR: reverse transcriptase-polymerase chain reaction; siRNA: small interference RNA; TNF-α: tumor necrosis factor-α.

## Competing interests

The authors declare that they have no competing interests.

## Authors' contributions

SS conceived this study and made substantial contributions to the study design and to writing the manuscript. SS also acquired and interpreted the data. YA and KA performed data acquisition, statistical analysis, and interpretation of data. KM participated in the design of the study and finalized the manuscript. YK and TK participated in the design of the study and performed the statistical analysis. CM performed the histologic analyses. AI carried out the *in vitro *assay of *ADAMTS5 *siRNA.
